# Supplementary material for: Development of a Microsphere-Based Immunoassay Authenticating A2 Milk and Species Purity in the Milk Production Chain
Source: Molecules. 2022 May 17;27(10):3199. doi: 10.3390/molecules27103199 (PMC9144198; doi:10.3390/molecules27103199)
Supplement: Supplementary file 1 [file molecules-27-03199-s001.zip › molecules-1682333-supplementary.pdf]

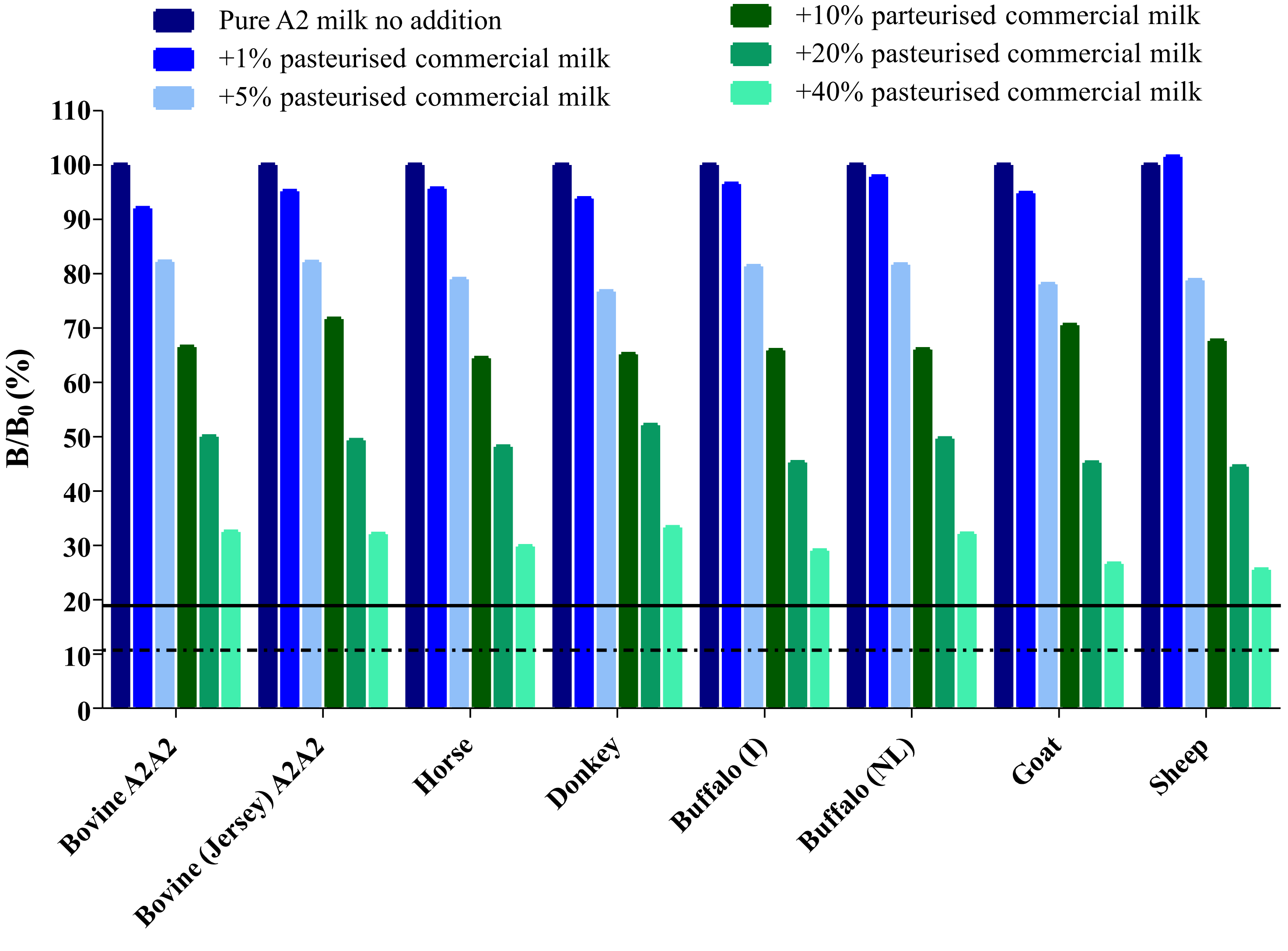

Figure S1. Average inhibition responses for the addition of 0-40% pasteurized A1A2 bovine milk to pure A2A2 milk from sheep, horse, buffalo, goat, donkey and cow. The black line indicates the average response for the pure pasteurized A1A2 bovine milk, while the black dotted line indicates the average response of a pure A1A1 milk sample.
